# Conformation-Driven Bilayer Nanocarriers for Anthocyanins Using Shell Polysaccharides: Stabilization Mechanisms and Enhanced In Vitro Lipid-Lowering Activity

**DOI:** 10.3390/molecules31101634

**Published:** 2026-05-13

**Authors:** Chunting Zhu, Jing Xu, Yunmei Ma, Yue Mi, Xing Yang, Dongfang Shi, Kai Song

**Affiliations:** 1 School of Life Science, Changchun Normal University, Changchun 130032, China; zct15948840694@163.com (C.Z.); xujingl@yeah.net (J.X.); yunmei0205@163.com (Y.M.); 18097730680@163.com (Y.M.); yangyx0816@163.com (X.Y.); 2 Institute of Innovation Science and Technology, Changchun Normal University, Changchun 130032, China

**Keywords:** blueberry anthocyanins, bilayer nanocarriers, gum arabic, carrageenan, lipid-lowering activity in vitro

## Abstract

Blueberry anthocyanins (BAs) exhibit strong antioxidant and lipid-regulating activities; however, their chemical instability and low oral bioavailability limit their practical application. In this study, two plant-based bilayer nanocarriers were developed using soybean lecithin as the lipid core and gum arabic (GA) or carrageenan (CGN) as the shell polysaccharide. The optimized systems achieved encapsulation efficiencies of 79.7% and 81.9%, respectively. Structural analyses showed that anthocyanins were stably incorporated into the carriers through multiple non-covalent interactions and transformed from a crystalline to an amorphous state. The two shell polysaccharides exhibited distinct conformation-dependent protective behaviors: GA provided better thermal protection, whereas CGN showed superior resistance to light, metal ions, ascorbic acid, and simulated intestinal digestion. After INFOGEST digestion, anthocyanin retention in the intestinal phase was 47% and 51% for the GA- and CGN-coated systems, respectively, and antioxidant activity was better preserved than in the free anthocyanin group. In an oleic-acid-induced HepG2 lipid accumulation model, the CGN carrier showed good biocompatibility and significantly enhanced the lipid-lowering effect of anthocyanins, with the most pronounced reduction in intracellular triglycerides. These results indicate that the CGN carrier has considerable potential for maintaining anthocyanin stability, modulating digestive behavior, and enhancing biological efficacy, and provide a reference for the design of plant-based delivery systems for bioactive ingredients.

## 1. Introduction

Anthocyanins (ACNs) are water-soluble natural pigments widely distributed in colored plant tissues such as blueberries. Their characteristic flavylium cation skeleton endows them with remarkable free-radical-scavenging capacity and diverse biological activities [1,2,3,4,5]. A growing body of epidemiological and in vitro evidence has demonstrated that blueberry anthocyanins (BAs), especially cyanidin-3-O-glucoside (C3G), are effective in suppressing oxidative stress, regulating lipid metabolism, and alleviating lipotoxic injury, highlighting their promise for functional food interventions [6,7,8,9,10,11,12,13,14]. However, the practical application of ACNs is hindered by pronounced chemical fragility. Under environmental stresses such as pH fluctuations [15,16,17,18], light [19], thermal processing [20,21], metal ions [22,23], and redox conditions [24], ACNs are highly susceptible to glycosidic bond cleavage and structural degradation. During oral delivery, the harsh gastrointestinal (GI) environment further results in extremely low bioavailability. Therefore, developing carrier systems capable of protecting ACNs’ integrity, improving digestive tolerance, and enhancing intracellular delivery efficiency is essential for translating their functionality into practical use.

Among various delivery strategies, lecithin-based liposomes have become attractive matrices for encapsulating bioactive compounds owing to their natural biocompatibility and amphiphilic self-assembly behavior [25,26,27,28,29,30,31,32]. Nevertheless, pure liposomal vesicles are prone to rupture and leakage in complex food matrices or GI microenvironments because of insufficient interfacial mechanical strength, temperature fluctuations, and competitive displacement by bile salts, which greatly compromises their protective performance [33,34]. To overcome these limitations, constructing bilayer composite carriers with a lipid core and a polysaccharide shell has emerged as an effective strategy to reinforce the interfacial barrier. Through electrostatic adsorption onto the liposomal surface, the polysaccharide shell forms a polyelectrolyte network that provides steric hindrance and markedly slows the penetration of external degrading factors [32,35,36].

Among plant-derived polysaccharide wall materials, gum arabic (GA), a highly branched natural heteropolysaccharide [37,38,39,40,41], and carrageenan (CGN), a linear anionic marine polysaccharide rich in sulfate groups [42,43,44,45,46,47], have attracted particular interest because of their markedly different molecular conformations and charge characteristics. Recent studies have preliminarily shown that polysaccharide–lipid composite systems can improve ACNs’ encapsulation stability, and that orally modified particles may enhance in vitro lipid-lowering activity [48,49,50,51,52,53]. However, important gaps remain. First, systematic comparative studies are lacking on how polysaccharides with distinct molecular conformations and charge densities, such as highly branched GA and linear sulfated CGN, regulate liposome assembly behavior and interfacial defense mechanisms. Specifically, although previous studies have shown that gum arabic-coated liposomes can improve the stability of blueberry anthocyanins [54] and that different polysaccharide wall materials influence the digestive release and bioaccessibility of anthocyanins [36], few studies have conducted a head-to-head comparison of highly branched GA and linear sulfated CGN on the same lecithin core. Second, current work rarely links the complete chain of evidence from nanoscale assembly mechanisms to extreme-environment tolerance, gastrointestinal fate, and cell-level terminal efficacy. For functional delivery systems intended to target lipid metabolism disorders, the translational value is substantially weakened if terminal effects are not validated in relevant physiological or pathological cell models.

**Figure 1:**
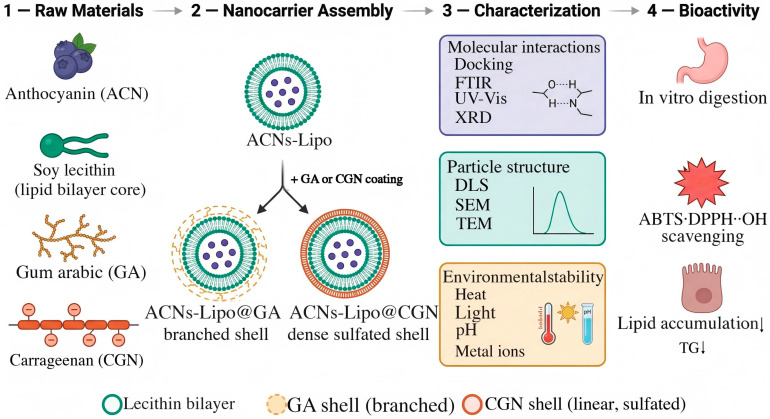
Construction strategy and research workflow of plant-based bilayer nanocarriers for blueberry anthocyanins. Abbreviations: ACNs, anthocyanins, defined above as BAs; GA, gum arabic; CGN, carrageenan; ACNs-Lipo, blueberry anthocyanin-loaded liposomes; ACNs-Lipo@GA, gum arabic-coated blueberry anthocyanin-loaded liposomes; ACNs-Lipo@CGN, carrageenan-coated blueberry anthocyanin-loaded liposomes; FTIR, Fourier-transform infrared spectroscopy; UV-Vis, ultraviolet-visible spectroscopy; XRD, X-ray diffraction; DLS, dynamic light scattering; SEM, scanning electron microscopy; TEM, transmission electron microscopy; ABTS, 2,2′-azino-bis(3-ethylbenzothiazoline-6-sulfonic acid); DPPH, 2,2-diphenyl-1-picrylhydrazyl; TG, triglycerides.

Accordingly, blueberry anthocyanins (BAs) were selected as the core bioactive compounds and used in all subsequent experiments; for convenience, they are hereafter referred to as anthocyanins (ACNs). Soybean lecithin was used as the lipid core, and GA or CGN was introduced to construct two plant-based bilayer nanodelivery systems, namely ACNs-Lipo@GA and ACNs-Lipo@CGN. After systematically optimizing the preparation process, molecular docking and multispectral techniques were employed to elucidate their molecular interactions and assembly mechanisms. The barrier effects of the two polysaccharide shells were comprehensively compared under heat, light, pH, metal-ion, and simulated gastrointestinal digestion stresses. In addition, antioxidant and lipid-lowering activities were validated using an oleic-acid-induced HepG2 lipid accumulation model. This study aimed to clarify how shell-polysaccharide conformation governs the physicochemical stability and biological efficacy of nanodelivery systems, thereby providing a theoretical basis for developing plant-based functional foods targeting lipid metabolic disorders. The overall experimental framework and technical workflow are presented in Figure 1.

## 2. Results

### 2.1. Process Optimization and Encapsulation Performance of Plant-Based Nanocarriers

#### 2.1.1. Single-Factor Screening

**Figure 2:**
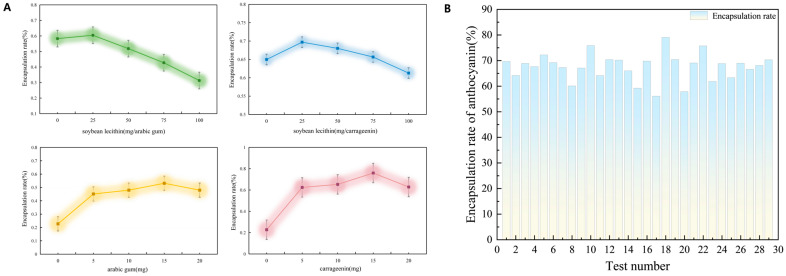
Process optimization and encapsulation performance of anthocyanin-loaded bilayer nanocarriers. (**A**) Effects of single factors on encapsulation efficiency; (**B**) response surface optimization and reproducibility validation.

Single-factor experiments systematically evaluated the effects of lecithin dosage, ACNs dosage, polysaccharide dosage, and total processing time (including ultrasonication and subsequent magnetic stirring) on the encapsulation efficiency (EE) of the two systems (Figure 2A). The EE of both ACNs-Lipo@GA and ACNs-Lipo@CGN showed a similar nonlinear dose-dependent trend with increasing component content, first increasing and then decreasing. This indicates that, under the single-factor experimental conditions, an appropriate wall-material ratio facilitates construction of a dense composite interface, whereas excessive addition (e.g., lecithin > 25 mg or polysaccharide > 15 mg) tends to saturate the interface, causing steric repulsion or particle flocculation and thereby weakening core retention. In addition, 30 min was the optimal total processing time (including ultrasonication and subsequent magnetic stirring) for promoting self-assembly between soybean lecithin and polysaccharides, whereas excessive shearing beyond this point likely disrupted the vesicle structure. Under identical conditions, the CGN system showed slightly greater encapsulation potential than the GA system, which may be attributed to the stronger electrostatic driving force provided by the high density of sulfate groups in CGN.

#### 2.1.2. Response Surface Optimization and Validation

Based on the single-factor screening results, a four-factor, three-level Box–Behnken design (BBD) was used to establish response surface regression models (see Supplementary Figure S1). Analysis of variance (ANOVA) showed that the quadratic regression models for both systems were highly significant (*p* < 0.001), while the lack-of-fit terms were not significant (*p* > 0.05), indicating good model fit and accurate prediction of interactions among multiple components.

For the ACNs-Lipo@GA system, ACNs dosage and total processing time (including ultrasonication and subsequent magnetic stirring) showed an extremely significant interaction (*p* < 0.01). The optimized preparation parameters were 25 mg lecithin, 0.30 g ACNs (lyophilized extract powder; actual amount added per batch), 20 mg GA, and 30 min homogenization, under which the measured EE was 79.70%, with a deviation of <2% from the predicted value. For ACNs-Lipo@CGN, lecithin dosage was the dominant factor affecting EE. The optimal parameters were 50 mg lecithin, 0.20 g ACNs (lyophilized extract powder; actual amount added per batch), 15 mg CGN, and 40 min homogenization, resulting in an EE of 81.87% (Figure 2B). Multi-batch reproducibility tests showed EE fluctuations of <2%, confirming process stability.

### 2.2. Molecular Interaction Mechanisms and Microstructural Analysis

#### 2.2.1. Molecular Docking and Multispectral Characterization

**Figure 3:**
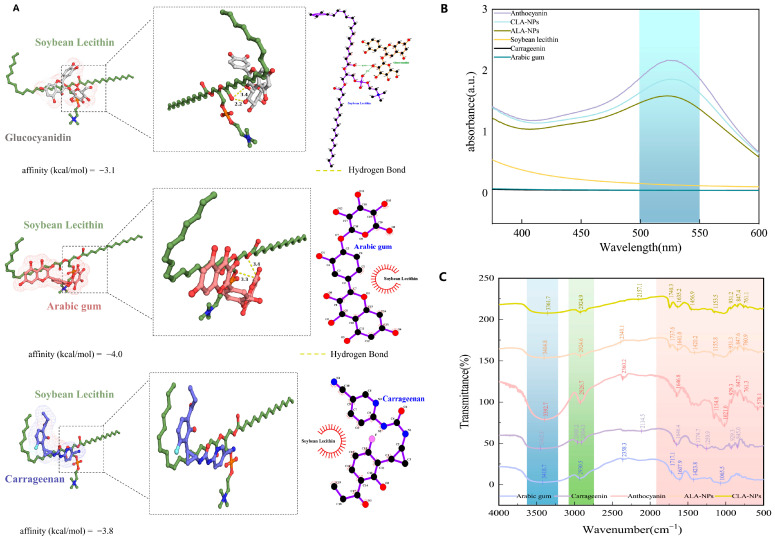
Molecular interactions between anthocyanins and carrier components and multispectral characterization. (**A**) Molecular docking models among C3G, GA, CGN, and soybean lecithin; (**B**) UV–Vis spectra of individual components and composite systems; (**C**) FTIR spectra. Abbreviations: C3G, Glucocyanidin; GA, Arabic gum; CGN, Carrageenan; CLA-NPs, ACNs-Lipo@GA; ALA-NPs, ACNs-Lipo@CGN; UV-Vis, ultraviolet-visible spectroscopy; FTIR, Fourier-transform infrared spectroscopy.

To clarify the molecular driving forces underlying the high encapsulation efficiency, molecular docking and multispectral characterization were combined for cross-validation. The docking results (Figure 3A) suggested that the anthocyanin monomer C3G may interact with the polar head groups of lecithin and polysaccharide chains through hydrogen bonding, with binding energies of approximately −3.1 to −4.0 kcal/mol. This indicates a certain binding tendency, although the overall binding strength remained moderate to weak. Meanwhile, the aromatic ring structure of C3G may interact mainly with the hydrophobic regions of the lipids through hydrophobic interactions. Notably, the dense sulfate groups on the CGN chains may enhance electrostatic adsorption with positively charged components, thereby facilitating interfacial complexation and coating. These molecular docking results should be interpreted only as qualitative indications of potential interaction modes; their explanation for the relatively high encapsulation efficiency should be considered together with the multispectral, particle-size, and morphological evidence.

Multispectral characterization supported these observations. The UV–Vis spectra (Figure 3B) showed that the characteristic absorption peak of anthocyanins at 520 nm did not shift markedly after encapsulation, although the absorption intensity decreased substantially. This indicates that the chromophore structure was preserved while anthocyanins were effectively embedded within the carriers. In the Fourier-transform infrared (FTIR) spectra (Figure 3C), the O–H stretching band at approximately 3400 cm^−1^ in the composite systems broadened and shifted toward lower wavenumbers, suggesting enhanced intermolecular hydrogen bonding. In addition, in the CGN system, the S=O characteristic band at approximately 1240 cm^−1^ shifted and overlapped, suggesting that sulfate groups may participate in interfacial interactions. This finding can be regarded as indirect evidence of electrostatic complexation but should not be considered direct proof.

#### 2.2.2. Particle Size Distribution, Phase Transition, and Morphology

**Figure 4:**
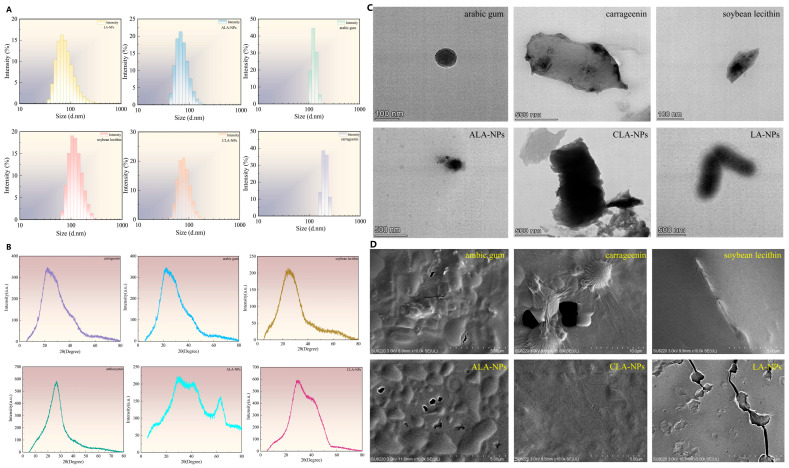
Particle size distribution, crystalline characteristics, and microscopic morphology of bilayer nanocarriers. (**A**) Particle size distribution and PDI of nanocarrier systems; (**B**) XRD phase analysis; (**C**) TEM observation of internal fine structure; (**D**) SEM analysis of surface morphology. Abbreviations: LA-NPs, ACNs-Lipo; C3G, Glucocyanidin; GA, Arabic gum; CGN, Carrageenan; CLA-NPs, ACNs-Lipo@GA; ALA-NPs, ACNs-Lipo@CGN; UV-Vis, ultraviolet-visible spectroscopy; FTIR, Fourier-transform infrared spectroscopy; PDI, Polydispersity Index; XRD, X-ray diffraction; SEM, scanning electron microscopy; TEM, transmission electron microscopy.

The multiple non-covalent interactions influenced the macroscopic physicochemical state of the carriers. Dynamic light scattering (DLS) results (Figure 4A) showed that the hydrodynamic diameters of ACNs-Lipo, ACNs-Lipo@GA, and ACNs-Lipo@CGN were 101.6, 116.4, and 105.3 nm, respectively. The increase in particle size after polysaccharide coating indicates that the polysaccharides were indeed deposited on the liposome surface and formed an outer coating layer. Among them, the CGN system showed a PDI of 0.256, suggesting a relatively narrower particle-size distribution, whereas the GA system showed a PDI of 0.312, indicating a relatively broader but still acceptable distribution. This difference may be related to the heterogeneous and flexible hydration layer formed by highly branched GA on the particle surface. Because zeta potential was not measured in this study, any inference regarding interfacial charge variation should be regarded as speculative rather than direct evidence.

The ACNs extract used in this study contained a total anthocyanin content of approximately 0.28 mg/g (expressed as cyanidin-3-glucoside equivalents), consistent with previously characterized blueberry extracts of similar origin [55,56]. The XRD pattern of the extract prior to encapsulation showed characteristic crystalline peaks (Figure 4B), which disappeared upon liposomal encapsulation, indicating successful incorporation of anthocyanin components into the lipid bilayer structure regardless of their individual identities [57,58]. The X-ray diffraction (XRD) patterns (Figure 4B) showed that free BAs exhibited a sharp crystalline diffraction peak at 2θ = 27°, whereas this characteristic peak was markedly weakened or disappeared in both composite carriers and was replaced by a broad amorphous halo. This result indicates that anthocyanins were transformed from their original crystalline state into an amorphously dispersed state within the lipid/polysaccharide matrix, suggesting relatively stable embedding within the carriers. However, this result reflects only a change in physical crystalline form and does not directly demonstrate improved bioavailability or the disappearance of specific degradation sites. SEM and TEM images (Figure 4C,D) further provided visual morphological observations. The composite carriers displayed well-defined and well-dispersed spherical contours. Under TEM, ACNs-Lipo@CGN showed a high-electron-density, core–shell-like morphology, whereas the shell outline of ACNs-Lipo@GA was relatively diffuse, suggesting the spatial looseness of the branched polysaccharide shell. No vesicle fusion was observed in either system, demonstrating good structural integrity.

### 2.3. Environmental Stability of Bilayer Nanocarriers

#### 2.3.1. Thermal and Photostability

**Figure 5:**
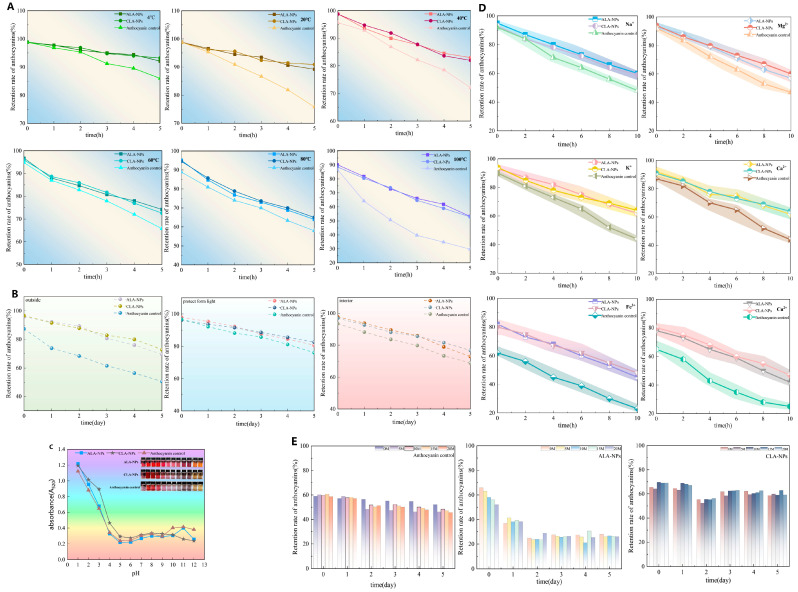
Protective effects of bilayer nanocarriers on anthocyanin stability during processing and storage. (**A**) Retention kinetics under thermal treatment at different temperatures; (**B**) stability under different light conditions; (**C**) stability across a pH gradient; (**D**) retention under metal-ion-induced oxidative stress; (**E**) retention under ascorbic acid-induced reducing stress. Abbreviations: CLA-NPs, ACNs-Lipo@GA; ALA-NPs, ACNs-Lipo@CGN.

ACNs control exhibited pronounced degradation sensitivity under environmental stress. In the thermal stability test (Figure 5A), the retention of ACNs control sharply declined to 29.77% after treatment at 100 °C for 5 h, whereas both nanocarrier systems showed significant thermal buffering effects (*p* < 0.05). Among them, ACNs-Lipo@GA, benefiting from the interfacial flexibility provided by its highly branched structure, effectively alleviated heat-induced volume expansion and particle collision, maintaining an ACNs retention rate of approximately 60% at 100 °C, which was significantly higher than that of ACNs-Lipo@CGN (52.89%, *p* < 0.05).

In contrast, the CGN system was superior in the photostability test (Figure 5B). After 5 days of outdoor natural light exposure, the retention rate of ACNs control declined to 50.48%, whereas ACNs-Lipo@CGN, owing to its dense linear macromolecular network, formed an efficient photon-shielding barrier and retained as much as 80% of ACNs, significantly outperforming the GA system (70%, *p* < 0.05). These findings suggest that differences in shell-polysaccharide conformation lead to differentiated physical defense mechanisms against distinct energy forms, namely heat and light.

#### 2.3.2. Stability Against pH, Metal Ions, and Ascorbic Acid

The pH stability test (Figure 5C) showed that all three groups were relatively stable under acidic conditions (pH 1.0–5.0) but underwent rapid ring opening and degradation in alkaline media (pH > 7.0). Nevertheless, both bilayer carriers retained ACNs significantly better than the free group (*p* < 0.05) over pH 7.0–12.0, and the CGN system exhibited stronger alkali resistance.

Metal ions accelerated the oxidative degradation of ACNs. As shown in Figure 5D, co-incubation with Fe^3+^ and Cu^2+^ for 10 h reduced the retention of ACNs control to approximately 23%, whereas ACNs-Lipo@CGN still maintained retention above 48% under these pro-oxidant ion stresses, significantly outperforming ACNs-Lipo@GA (*p* < 0.05). This was likely due to the dense polyanionic sulfate groups on CGN, which may create a negatively charged interfacial region that could electrostatically hinder the approach of multivalent cations (e.g., Fe^3+^ and Cu^2+^), thereby reducing their accessibility to the lipid core and limiting metal-catalyzed oxidative degradation of ACN.

A similar trend was observed in the ascorbic acid stability test (Figure 5E). Under a strongly reducing environment containing 20 mmol/L ascorbic acid, ACNs control were severely degraded, with retention decreasing to 45.56%, whereas ACNs-Lipo@CGN preserved 59.13% of the active compounds by virtue of its compact interfacial barrier. Taken together, the GA system appears more suitable for high-temperature processed foods, while the CGN system shows broader compatibility in ion-rich matrices and high-light-exposure scenarios.

### 2.4. In Vitro Gastrointestinal Fate and Antioxidant Activity

#### 2.4.1. Anthocyanin Retention Kinetics During Gastrointestinal Digestion

**Figure 6:**
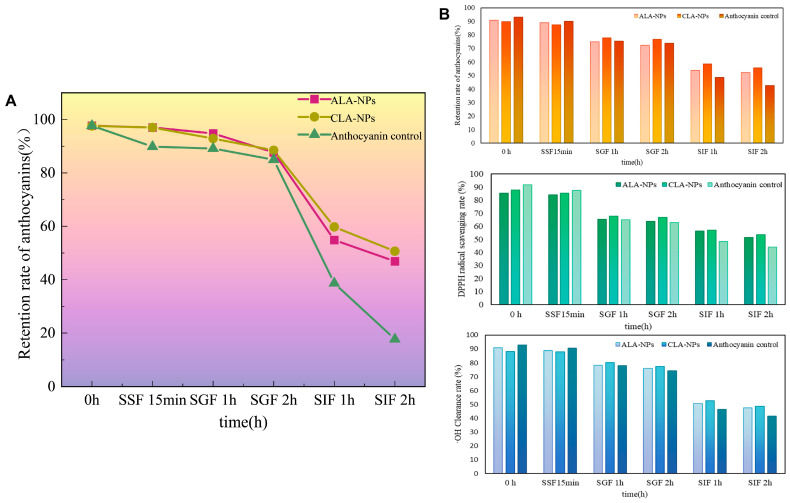
Bilayer nanocarriers improve digestive tolerance and preserve radical-scavenging activity. (**A**) Anthocyanin retention curves during INFOGEST simulated digestion; (**B**) ABTS^+^, DPPH, and hydroxyl radical scavenging capacities at the digestion endpoint. Abbreviations: CLA-NPs, ACNs-Lipo@GA; ALA-NPs, ACNs-Lipo@CGN; ABTS, 2,2′-azino-bis(3-ethylbenzothiazoline-6-sulfonic acid); DPPH, 2,2-diphenyl-1-picrylhydrazyl; SSF, simulated gastrointestinal digestion; SGF, gastric stage SIF, intestinal stage.

The INFOGEST static digestion model was used to track ACNs’ degradation in the GI tract (Figure 6A). During the oral (SSF, pH 7.0) and gastric (SGF, pH 3.0) digestion stages, free BAs underwent only limited degradation because of their short-term exposure to neutral to weakly acidic environments. By contrast, the encapsulated groups (ACNs-Lipo@GA and ACNs-Lipo@CGN) benefited from the stability of the lipid bilayer under gastric acidic conditions and the steric hindrance of the polysaccharide shell against pepsin, allowing all three samples to maintain retention rates above 85%.

After entering the intestinal stage (SIF), the ACNs control underwent marked degradation under the combined effects of weakly alkaline pH-induced structural transformation, bile-salt solubilization, and pancreatin, leaving only 17.68% retention after 2 h. In sharp contrast, at the end of the SIF stage, ACNs-Lipo@CGN and ACNs-Lipo@GA still retained 51% and 47% of ACNs, respectively. These results indicate that the bilayer carriers exerted both sustained-release and degradation-protective effects in the harsh intestinal microenvironment. The slightly superior performance of the CGN system may be associated with its highly crosslinked polyelectrolyte shell, which is more favorable for resisting competitive displacement of the internal phospholipid membrane by bile salts.

#### 2.4.2. Dynamic Changes in Antioxidant Activity After Digestion

The integrity of ACNs’ structure directly determines the retention of terminal bioactivity. As shown in Figure 6B, the ACNs control exhibited markedly reduced radical-scavenging capacity after the complete GI digestion process, with ABTS^+^, DPPH, and ·OH scavenging rates decreasing to approximately 42.59%, 44.05%, and 41.6%, respectively. In contrast, both nanodelivery systems maintained higher antioxidant activities at the digestion endpoint. Among them, ACNs-Lipo@CGN ranked first across all three assays, with scavenging rates of 55.67%, 53.7%, and 48.79%, respectively, demonstrating that the strategy of a physically shielding polysaccharide shell combined with a stabilizing lipid core effectively preserved ACNs in a highly active form until they reached the intestinal absorption window.

### 2.5. Cell Compatibility and In Vitro Lipid-Lowering Efficacy

#### 2.5.1. Cell Compatibility

**Figure 7:**
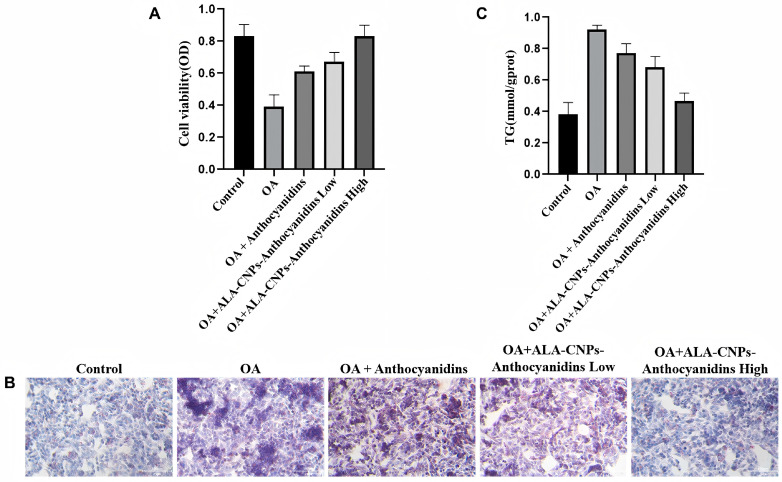
In vitro intervention effects of bilayer nanocarriers on OA-induced lipid accumulation in HepG2 cells. (**A**) CCK-8 assay defining the safe concentration window; (**B**) Oil Red O staining of intracellular lipid droplets (200× magnification); (**C**) quantitative analysis of intracellular triglycerides (TG). Abbreviations: ALA-NPs, ACNs-Lipo@CGN; OA, oleic acid.

To ensure the safety of the nanocarrier for cell experiments, the cytotoxicity threshold of ACNs-Lipo@CGN toward HepG2 cells was first evaluated using the CCK-8 assay (Figure 7A). Within an anthocyanin-equivalent concentration range of 0–500 μg/mL, cell viability remained above 90% and did not differ significantly from that of the blank control (*p* > 0.05), indicating good biocompatibility of the CGN nanocarrier. Based on this result, 300 μg/mL (low dose) and 500 μg/mL (high dose) were selected for subsequent efficacy validation.

#### 2.5.2. Inhibition of OA-Induced Lipid Accumulation

Treatment of HepG2 cells with 0.5 mmol/L oleic acid (OA) for 24 h successfully established a hepatic lipid accumulation model. Oil Red O staining (Figure 7B) showed that a large number of densely distributed neutral lipid droplets formed in the cytoplasm of the model group (OA). In contrast, after intervention with high-dose ACNs-Lipo@CGN, both the number and projected area of intracellular lipid droplets were markedly reduced, and cell morphology approached that of the blank control, suggesting a strong lipid-lowering effect that was visually superior to that of the free BAs treatment.

Absolute quantification of intracellular TG (Figure 7C) provided further statistical support. Compared with the model group, high-dose free BAs reduced TG content only to 77.3% of the model level, indicating relatively limited efficacy. By contrast, high-dose ACNs-Lipo@CGN reduced intracellular TG content to 46.9% of the model level, representing a further reduction of more than 39% compared with the free anthocyanin group (*p* < 0.05). These results indicate that nanoencapsulation enhanced the anti-lipotoxic effect of anthocyanins in HepG2 cells, although whether this effect is specifically related to improved cellular uptake efficiency remains to be verified by further uptake/endocytosis experiments.

## 3. Discussion

### 3.1. Regulatory Role of Shell Polysaccharide Conformation in Nanocarrier Self-Assembly and Encapsulation Efficiency

This study demonstrated that both GA and CGN could cooperate with soybean lecithin to construct anthocyanin-loaded bilayer nanodelivery systems with high retention capability [54,59], while their markedly different macromolecular conformations substantially regulated interfacial properties and encapsulation performance [60]. Cyanidin-3-glucoside (C3G), as the predominant anthocyanin in blueberry extracts and the most extensively studied congener, was used as a reference compound to illustrate the mechanistic basis of the observed effects [56,61,62]. It is acknowledged that the ACNs control extract employed in this study contains multiple anthocyanin species, and their synergistic contributions to the observed bioactivities cannot be excluded. Through amphiphilic self-assembly, soybean lecithin provided an initial hydrophobic/hydrophilic segregated microenvironment for ACNs [63]. However, pure liposomal interfacial membranes are mechanically weak and susceptible to vesicle fusion [64]. Introducing a polysaccharide shell is therefore an effective means of barrier reinforcement, but the present comparison revealed distinct conformation-mediated interfacial behaviors [65].

As a highly branched natural heteropolysaccharide, GA formed a thick, highly hydrated, and flexible steric layer on the particle surface owing to its excellent surface activity. This relatively loose hydrated shell conferred good colloidal dispersibility on the system but had a relatively limited capacity for dense entrapment of the core molecules [66]. By contrast, CGN is a linear anionic polysaccharide rich in sulfate groups. It is extracted from red algae and is widely used in the food, pharmaceutical, and industrial fields. According to its structural characteristics, carrageenan is generally classified into several types, including κ-carrageenan, ι-carrageenan, and λ-carrageenan; among them, κ-carrageenan has strong gel-forming ability and is widely used for thickening, stabilization, and emulsification in foods. Under the preparation conditions (pH 4.0), CGN chains in an extended-coil conformation may undergo electrostatic adsorption and polyelectrolyte crosslinking with lecithin polar head groups (phosphatidylcholine is zwitterionic, and its surface charge state at pH 4.0 requires further verification) and the flavylium cations of anthocyanins, thereby promoting the formation of a denser three-dimensional network around the lipid core [59,67]. This coating behavior, jointly driven by chain conformation, electrostatic complexation, and interchain entanglement, provides a physicochemical explanation for the higher encapsulation efficiency of the CGN system (EE = 81.87%) compared with the GA system (EE = 79.70%) [54].

### 3.2. Multiple Non-Covalent Crosslinking and Phase Transformation: The Physicochemical Basis of Microstructural Stability

The assembly mode driven by multiple cooperative interactions constitutes the physicochemical basis for the macroscopic stability of plant-based nanodelivery systems. Multispectral analysis and molecular docking jointly confirmed that hydrogen bonding, hydrophobic association and electrostatic interactions built a dense non-covalent network within the system [50]. This agrees with the ACNs encapsulation mechanism observed by Cui et al. [36] in protein–polysaccharide complexes, while the present study further clarifies the specific contribution of sulfate-rich polyanions.

More importantly, these strong intermolecular interactions directly triggered a phase transformation of the active compounds. The complete disappearance of the characteristic crystalline peak of ACNs control in the XRD patterns indicates a full conversion from a thermodynamically unstable crystalline state to an amorphous molecularly dispersed state confined within the matrix [68]. This lattice disruption is highly relevant for delivery: on the one hand, the amorphous state removes preferential degradation sites present on crystal surfaces and greatly reduces reactivity under environmental stress; on the other hand, the highly dispersed amorphous form lowers the lattice-energy barrier for transmembrane release and thereby provides a thermodynamic basis for improved intestinal absorption and bioavailability [68].

The interaction between anthocyanins and the liposomal carrier may involve multiple modes, including association with the lipid bilayer interface, partial intercalation into the hydrophobic core of the bilayer, or partitioning into the aqueous interior, depending on the polarity and glycosylation state of individual ACNs species [69,70,71]. The precise localization of ACNs within the liposomal structure warrants further investigation using techniques such as fluorescence quenching or small-angle X-ray scattering.

Moreover, polysaccharide conformation also continued to regulate microscopic morphology: according to TEM observations, the linear and highly charged CGN formed a relatively continuous coating-like structure [59], whereas the highly branched GA endowed the particles with excellent interfacial flexibility, a physical feature that played a key positive buffering role against subsequent thermal stress [72] ].

### 3.3. Physical Barrier and Microenvironment Reconstruction: Defense Across Extreme Environments and the Gastrointestinal Barrier

In the absence of protection, the flavylium cation of ACNs is highly vulnerable to irreversible degradation under heat, light, pH fluctuation, and redox attack [73]. The bilayer nanocarriers developed in this work exhibited excellent multidimensional stress resistance, the core mechanism of which lies in the synergy between physical barrier isolation and microenvironmental polarity reconstruction [63,74].

Regarding differentiated defense mechanisms, the GA system showed the best thermal stability at 100 °C [72]. This not only supports the findings of Ali et al. [75] on the ability of GA to stabilize high-temperature emulsions, but also extends that concept to the delivery of ACNs small molecules, namely that the interfacial flexibility of the GA shell can effectively absorb thermal expansion shocks and prevent vesicle rupture. By contrast, the CGN system, owing to its dense linear network and electrostatic repulsion from sulfate groups, showed clear advantages in blocking photon penetration, chelating/repelling pro-oxidant metal ions, and resisting the strong reducing consumption caused by ascorbic acid [67,76].

During INFOGEST digestion, free ACNs underwent severe structural collapse under the weakly alkaline conditions and enzymatic action in SIF. In contrast, the bilayer carriers, especially the CGN system, not only slowed digestive-fluid penetration but also strongly resisted the competitive displacement of the lipid bilayer by bile salts because of the negatively charged polyelectrolyte shell [77]. The 51% retention achieved at the end of the SIF stage is substantially higher than that commonly reported for monolayer liposomes, which is typically below 30% [78]. This dual function of sustained release and degradation protection is fundamental to ensuring that ACNs reach the intestinal absorption site in a highly active form and maintain antioxidant activity at the digestion endpoint [63]. It is acknowledged that the inclusion of uncoated ACNs-Lipo as an additional control group would allow direct quantification of the incremental contribution of the polysaccharide shell to digestive stability. This represents a limitation of the present study and warrants further investigation. Nevertheless, the superior stability of polysaccharide-coated liposomes over ACNs control observed here is consistent with the established protective role of polysaccharide coatings in liposomal delivery systems [79,80,81,82].

### 3.4. Cascade Amplification from Structure to Stability to Efficacy: Enhanced Intracellular Relief of Lipotoxicity

The ultimate mission of plant-based nanocarriers is to translate improved macroscopic physicochemical stability into amplified biological efficacy at the cellular level. Although free ACNs possess lipid-regulating potential, their intrinsic hydrophilicity severely limits transmembrane permeability, often preventing intracellular concentrations from reaching the effective intervention threshold [73]. The cell experiments in the present study clearly demonstrate that bilayer nanoencapsulation successfully overcame this pharmacokinetic bottleneck [54].

First, the high similarity between the soybean lecithin core and the phospholipid layer of the cell membrane may facilitate efficient cellular uptake of the nanoparticles, potentially through membrane fusion or endocytosis [51]. Second, the polysaccharide shell may not only serve as an extracellular barrier against oxidative attack but may also help slow the rapid degradation of anthocyanins after cellular entry [54]. Although the present study does not provide direct experimental evidence for endocytosis, lysosomal protection, or intracellular sustained release, this proposed delivery logic—extracellular protection, membrane-affinitive uptake, and possible intracellular protection—is consistent with the observation that the nanocarriers were more effective than free anthocyanins in the OA-induced HepG2 model. Notably, the superior lipid-lowering performance of the CGN system strongly supports the hypothesis that higher structural integrity drives greater biological efficacy: a higher encapsulation efficiency provides greater payload capacity, while a denser barrier leads to higher digestive retention, and together these advantages determine superior terminal efficacy [77]. Because the entire system was built from food-grade ingredients (lecithin, CGN), its excellent cell compatibility further supports future translation into functional foods [83]. This rational design paradigm based on polysaccharide conformation offers valuable theoretical and practical guidance for targeted delivery systems for plant polyphenols [84]. Although a blank carrier control (empty liposomes without anthocyanins) was not included in the present study, previous reports have indicated that phosphatidylcholine-based liposomes at comparable concentrations do not significantly interfere with lipid metabolism in HepG2 cells [85]. Nevertheless, the inclusion of vehicle controls represents a limitation that should be addressed in future mechanistic studies.

It should be noted that the anthocyanin retention measured using the INFOGEST model in this study reflects only bioaccessibility, namely the fraction released from the food matrix and potentially available for absorption, and does not equate to bioavailability. Bioavailability also involves absorption by intestinal epithelial cells, metabolism, transport, and other processes, which were not evaluated in this study. Therefore, improved bioaccessibility does not necessarily imply improved bioavailability. The HepG2 cell results only demonstrate that anthocyanins can ameliorate lipid accumulation after entering hepatic cells, but they do not directly demonstrate increased intestinal absorption efficiency. Future studies should further validate the actual bioavailability of this nanocarrier using Caco-2 monolayer cell models and animal models.

This study has several limitations. First, the composition of the anthocyanin extract was not fully characterized by HPLC. Second, the intestinal absorption of anthocyanins was not evaluated using a Caco-2 cell model. Third, direct evidence for the intracellular uptake mechanism of the nanocarriers was not provided. Therefore, the discussion regarding in vivo bioavailability and absorption mechanisms in this manuscript remains speculative and should be validated in future studies using in vivo animal models and intestinal transport models.

## 4. Materials and Methods

### 4.1. Materials and Reagents

Fresh blueberries (*Vaccinium* spp.) were obtained from the Heyun Blueberry Plantation Base, Tonghua, Jilin Province, China. Soybean lecithin (phosphatidylcholine content ≥ 98%), GA, CGN, pepsin (≥250 U/mg), pancreatin (≥4 × USP), porcine bile salts, and OA were purchased from Beijing Solarbio Science & Technology Co., Ltd. (Beijing, China) or Shanghai Yuanye Bio-Technology Co., Ltd. (Shanghai, China). ABTS, DPPH, and hydroxyl radical scavenging assay kits were obtained from commercial suppliers. The human hepatocellular carcinoma cell line HepG2 was provided by the Cell Bank of the Chinese Academy of Sciences (Shanghai, China). CCK-8 cell viability kits, TG assay kits, and Oil Red O staining solution were purchased from Nanjing Jiancheng Bioengineering Institute (Nanjing, China). Ascorbic acid, metal salts (NaCl, KCl, MgCl_2_, CaCl_2_, FeCl_3_, and CuCl_2_), and other reagents were of analytical grade. Ultrapure water (resistivity ≥ 18.2 MΩ·cm) was used in all experiments.

### 4.2. Extraction of Blueberry Anthocyanins

Fresh blueberries without mechanical damage were selected, washed, drained, and homogenized. A 30 g aliquot of blueberry homogenate was accurately weighed and extracted at a solid-to-liquid ratio of 1:30 (g/mL) using an extraction solvent consisting of absolute ethanol containing 0.1% HCl. Ultrasound–microwave synergistic-assisted extraction was performed using an XO-SM100 ultrasound–microwave combined reaction system (Nanjing Xianou Instruments Manufacturing Co., Ltd., Nanjing, China) at 60 °C for 30 min, with the ultrasound and microwave powers set at 300 W and 300 W, respectively. The extraction was repeated twice, and the extracts were combined. After extraction, the mixture was centrifuged using an L550 centrifuge at 4 °C and 5000 r/min (2348× *g*, Sigma 3-30K) for 10 min (Xiangyi Centrifuge Instrument Co., Ltd., Changsha, Hunan, China) to remove insoluble impurities, and the supernatant was collected. The supernatant was then concentrated using an RV10 rotary evaporator (IKA, Staufen, Germany) at 40 °C under reduced pressure to remove ethanol. After the concentrate was adjusted to a uniform volume, it was vacuum freeze-dried using an LGJ-10 vacuum freeze dryer (Beijing Songyuan Huaxing Technology Development Co., Ltd., Beijing, China) to obtain blueberry anthocyanin (BAs) lyophilized powder [86]. The lyophilized powder was redissolved in ultrapure water, adjusted to pH 4.0 with citrate–sodium citrate buffer, and stored at 4 °C in the dark until use. Total anthocyanin content was determined by the pH differential method. Absorbance was measured at 520 and 700 nm using an HBS-1096ScanY full-wavelength microplate reader (Nanjing Detie Biotechnology Co., Ltd., Nanjing, China), and quantification was performed using cyanidin-3-O-glucoside (C3G) as the standard [87].

### 4.3. Construction and Process Optimization of Plant-Based Bilayer Nanocarriers

#### 4.3.1. Preparation of Nanocarriers

The bilayer nanocarriers were constructed in three steps using direct hydration combined with electrostatic deposition [82].

(1) Preparation of primary liposomes: soybean lecithin was dispersed in ultrapure water and magnetically stirred at room temperature for 30 min to ensure complete hydration, after which the pH was adjusted to 4.0 using citrate buffer. The suspension was placed in an ice bath and treated with a probe sonicator (Ningbo Scientz Biotechnology Co., Ltd., Ningbo, China) at an absolute output power of 600 W (5 s on/2 s off) for 40 min to obtain a homogeneous primary liposome suspension (Lipo).

(2) Anthocyanin loading: the ACNs solution was slowly added dropwise to the Lipo suspension and magnetically stirred at 25 °C for 40 min in the dark, allowing ACNs to be incorporated into the liposomal carrier to form the loaded core (ACNs-Lipo).

(3) Polysaccharide shell coating: The pre-sonicated GA or CGN solution was slowly added dropwise to the ACNs-Lipo suspension at a constant flow rate. In the final mixture, the initial volume ratio of anthocyanin solution, blank liposome suspension, and polysaccharide solution was 2:1:2; specifically, the anthocyanin solution and blank liposome suspension were first mixed at a 2:1 ratio to obtain ACNs-Lipo, which was then mixed with two volumes of polysaccharide solution. The system was maintained at pH 4.0 and magnetically stirred in the dark for 40 min to promote the deposition of polysaccharide molecules onto the liposome surface through electrostatic interactions, yielding the bilayer nanocarriers ACNs-Lipo@GA and ACNs-Lipo@CGN, respectively. All preparation steps were performed under light-protected conditions, and the obtained products were stored at 4 °C until use. Specifically, the CGN used in this study was κ-carrageenan (CAS 11114-20-8), with a molecular weight of 788.65764 Da, and the stock solution concentration was 1.5 mg/mL. The ultrasonic dispersion conditions were an ultrasonic power of 600 W, a treatment time of 40 min, and room temperature. During dropwise addition, the flow rate was set to 1.5 mL/min.

#### 4.3.2. Optimization by Response Surface Methodology

EE as the response value, single-factor experiments were first performed to investigate the effects of lecithin amount, ACNs amount, polysaccharide amount, and total processing time (including ultrasonication and subsequent magnetic stirring) on EE. On this basis, a four-factor, three-level BBD was applied to optimize the ACNs-Lipo@GA and ACNs-Lipo@CGN systems separately. Experimental data were analyzed using Design-Expert 13.0 through multivariate regression and ANOVA. A model with *p* < 0.01 and a lack-of-fit term with *p* > 0.05 was considered significant and acceptable. The optimal parameters were validated experimentally and used for all subsequent batches.

### 4.4. Determination of Encapsulation Efficiency

ACNs control content was determined by high-speed refrigerated centrifugation combined with the pH differential method, and EE was calculated accordingly. The nanocarrier suspension was centrifuged at 4 °C and 8000 r/min (6010× *g*) for 20 min, and the supernatant containing ACNs control was collected. Absorbance at 520 and 700 nm was measured using a UV–Vis spectrophotometer (Shanghai Spectrum Instruments Co., Ltd., Shanghai, China). Encapsulation efficiency was calculated as EE (%) = (*ACN*_0_ − *ACN*_u_)/*ACN*_0_ × 100, where *ACN*_0_ is the total mass of ACNs added to the system and *ACN*_u_ is the mass of ACNs control in the supernatant.

### 4.5. Molecular Docking Simulation

To reveal the binding mechanisms between ACNs and carrier components at the molecular level, AutoDock Vina (version 1.2.2) was used for molecular docking. C3G was selected as the ligand, while soybean lecithin (phosphatidylcholine, DPPC) and representative polysaccharide fragments (GA or CGN) were used as receptors. The three-dimensional structures of all ligands and receptors were obtained from the PubChem database and preprocessed with AutoDock Tools by adding hydrogens and charges. The grid box covered the receptor active region, with a grid spacing of 0.375 Å. The lowest binding energy (kcal/mol) of each complex was used to evaluate binding affinity, and hydrogen-bond networks and hydrophobic interactions at the binding interface were visualized in three dimensions using PyMOL (version 2.5).

DPPC (dipalmitoylphosphatidylcholine) was selected as a substitute for soybean lecithin in molecular docking mainly for the following reasons. The major component of soybean lecithin, accounting for approximately 70–80%, is phosphatidylcholine (PC), whose amphiphilicity and head-group charge are the primary driving forces for interactions between polysaccharides and lipids [88,89]. DPPC is a structurally defined, saturated representative PC molecule with well-validated crystal structure and force-field parameters, and it has been widely used as a model of plant phospholipids [90]. Using a single molecular species, DPPC, avoids competitive effects arising from different phospholipids in natural lecithin, such as PE and PI, as well as differences in fatty-acid chain unsaturation, thereby allowing clearer analysis of the binding modes between polysaccharides and PC head groups/hydrophobic tails.

For gum arabic (GA), a representative branched structural fragment was used for docking. GA is a highly branched hydrophilic anionic heteropolysaccharide with a backbone composed of β-(1→3)-D-galactopyranose and side chains linked through β-(1→6) bonds, mainly consisting of arabinofuranose, galactopyranose, rhamnopyranose, and uronic acids. For κ-carrageenan (CGN), the repeating unit consists of alternating α-(1→3)-D-galactose-4-sulfate and β-(1→4)-3,6-anhydro-D-galactose, with one sulfate group (SO_3_^−^) in each repeating unit. The CGN fragment used in this study contained eight repeating disaccharide units, corresponding to 16 monosaccharide units in total, with a molecular formula of C_24_H_36_O_25_S_2_^2−^ and a molecular weight of 788.66 Da, to reflect the linear sulfated characteristics of κ-carrageenan. The three-dimensional structures of GA and CGN were obtained from the PubChem database and preprocessed.

### 4.6. Characterization of Microstructure and Physicochemical Properties

(1) Multispectral analysis: freeze-dried samples of ACNs control, single wall materials (GA and CGN), lecithin (Lipo), and the two composite nanocarriers were collected. UV–Vis spectra were recorded over 380–600 nm. FTIR (Nicolet iS50, Thermo Fisher Scientific, Waltham, MA, USA) was performed over 400–4000 cm^−1^ using the KBr pellet method. Raman spectra were recorded over 400–2000 cm^−1^ (see Supplementary Figure S1).

(2) Particle size, zeta potential, and dispersity: the hydrodynamic diameter (Z-average), polydispersity index (PDI), and zeta potential of each system were measured at 25 °C using a Zetasizer Nano ZS (Malvern Instruments, Malvern, UK), with three replicates per sample.

(3) Morphology observation: surface morphology of the freeze-dried samples was observed by SEM (Carl Zeiss AG, Oberkochen, Germany) after gold sputtering (accelerating voltage, 5 kV), and internal fine structures were examined by TEM (Thermo Fisher Scientific, Waltham, MA, USA) after negative staining with 2% phosphotungstic acid for 1 min and natural drying.

(4) XRD (Bruker AXS GmbH, Karlsruhe, Germany): crystal phase analysis of freeze-dried samples was performed over 2θ = 5–45° at a scanning rate of 2 °/min and a step size of 0.02°.

### 4.7. Environmental Stability Evaluation

Dispersions of ACNs control, ACNs-Lipo@GA, and ACNs-Lipo@CGN at the same concentration were subjected to the following stress conditions. Each group contained three parallel samples. ACNs retention rate (RR) was used as the evaluation index and calculated according to the absorbance-based method described in Section 2.4.

(1) Thermal stability: samples were placed in water baths at 4, 20, 40, 60, 80, and 100 °C for 0–5 h, and aliquots were collected every hour.

(2) Photostability: Samples were stored for 0–5 days under dark conditions (aluminum-foil shielding), indoor scattered light, or outdoor natural light (approximately 8000 lux at noon on a sunny day), and sampled daily for analysis.

(3) pH stability: To systematically evaluate the effects of different pH environments on system stability, a series of buffer solutions with precise buffering capacity were used to establish a full-range pH gradient, rather than direct adjustment with hydrochloric acid (HCl) or sodium hydroxide (NaOH), to ensure stable pH values and reproducible results during the experiment. The buffer systems were classified according to pH range as follows: strongly acidic conditions (pH 1–3), 0.2 mol/L potassium hydrogen phthalate–HCl and 0.2 mol/L glycine–HCl; weakly acidic conditions (pH 4–6), 0.2 mol/L acetic acid–sodium acetate and 0.1 mol/L citric acid–sodium citrate; neutral conditions (pH 7), 0.2 mol/L phosphate buffer and 0.1 mol/L Tris–HCl; weakly alkaline conditions (pH 8–10), 0.05 mol/L borax–boric acid and 0.2 mol/L glycine–NaOH; and strongly alkaline conditions (pH 11–12), 0.1 mol/L sodium carbonate–sodium bicarbonate and 0.2 mol/L disodium hydrogen phosphate–NaOH. After the samples were mixed with the corresponding buffers, they were allowed to stand at room temperature for 1 h and then analyzed.

(4) Metal ion stability: samples were mixed with Na^+^, K^+^, Mg^2+^, Ca^2+^, Fe^3+^, or Cu^2+^ solutions (final concentration, 1 mmol/L each) and co-incubated in the dark for 0–10 h, with sampling every 2 h.

(5) Ascorbic acid stability: 0, 5, 10, 15, and 20 mmol/L ascorbic acid was added to the samples, which were incubated in the dark at room temperature for 2 h before analysis.

### 4.8. In Vitro Simulated Gastrointestinal Digestion and Antioxidant Activity

The INFOGEST method was used to perform in vitro simulated digestion experiments to evaluate the stability of nanoparticle-complex samples and free anthocyanin control samples under simulated digestive conditions. The experiment consisted of oral, gastric, and small-intestinal stages, with different simulated digestive fluids and enzymes used at each stage to mimic the human digestive process. All experiments were performed in a thermostatic shaker at 37 °C and 300 rpm. For sample preparation, 20 mL of nanoparticle-complex sample and 20 mL of free anthocyanin control sample were separately placed in 50 mL centrifuge tubes.

For digestive fluid preparation, the electrolyte stock solutions of simulated salivary fluid (SSF), simulated gastric fluid (SGF), and simulated intestinal fluid (SIF) were prepared in advance, stored at −20 °C, and equilibrated to 37 °C before use.

(1) Oral digestion stage (SSF): pre-equilibrated SSF was added to the sample at 3–4 times the sample volume. α-Amylase was added to a final concentration of 1000 U/mL, and CaCl_2_ was added to give a final Ca^2+^ concentration of 0.3 M. The pH was adjusted to 7.0 ± 0.1 using 1 mol/L HCl. The mixture was incubated at 37 °C and 300 rpm for 15 min. After incubation, the reaction was terminated by cooling in an ice bath, and the sample was immediately subjected to the gastric stage.

(2) Gastric digestion stage (SGF): pre-equilibrated SGF was added to the above system. Pepsin was added to a final concentration of 60,000 U/mL, and CaCl_2_ was added to give a final Ca^2+^ concentration of 0.3 M. The pH was adjusted to 3.0 using 1 mol/L HCl, and the volume was made up as required. The mixture was incubated at 37 °C and 300 rpm for 2 h. After incubation, the reaction was terminated by cooling in an ice bath, and the sample was immediately subjected to the small-intestinal stage.

(3) Small-intestinal digestion stage (SIF): pre-equilibrated SIF was added to the above system. Bile salts and pancreatin were added to achieve a final trypsin activity of 800 U/mL, and CaCl_2_ was added to give a final Ca^2+^ concentration of 0.3 M. The pH was adjusted to 7.0 using 1 mol/L NaOH, and the volume was made up as required. The mixture was incubated at 37 °C and 300 rpm for 2 h. After incubation, the reaction was terminated by cooling in an ice bath, and the samples were stored at 4 °C until analysis.

At the end of each digestion stage, samples were collected and centrifuged at 4 °C and 5000 r/min for 10 min, and the supernatants were used to determine ACNs retention. It should be noted that the anthocyanin bioaccessibility measured in this study, namely the proportion of anthocyanins released into the supernatant after digestion, was defined as the percentage of anthocyanins in the supernatant relative to the initial amount added, rather than the total retention of anthocyanins in the sample. Anthocyanins that remained bound to nanoparticles and were not released during digestion were removed with the precipitate and were not included in the calculation. Under the present centrifugation conditions, nanoparticles were completely sedimented, and anthocyanins in the supernatant represented the free form. Therefore, this index reflects the potential fraction of anthocyanins available for absorption after digestion. The supernatant collected at the end of the intestinal stage was further assayed for ABTS^+^, DPPH, and hydroxyl radical (·OH) scavenging according to the kit instructions. These three methods are based on electron-transfer mechanisms (ABTS^+^ and DPPH) and specific scavenging of hydroxyl radicals (·OH), respectively. Their combined use provides a more comprehensive assessment of antioxidant capacity. The results were expressed as percentage scavenging rate (%), namely the ability of each sample to scavenge the corresponding radical.

### 4.9. Cell Experiments and Verification of In Vitro Lipid-Lowering Activity

#### 4.9.1. Cell Culture and Biocompatibility Assessment

HepG2 cells were routinely cultured in DMEM complete medium containing 10% fetal bovine serum (FBS) and 1% penicillin–streptomycin in a humidified incubator at 37 °C with 5% CO_2_. The medium was replaced and cells were passaged every 2–3 days. Cell compatibility was evaluated using the CCK-8 assay. HepG2 cells in the logarithmic growth phase were seeded into 96-well plates at 5 × 10^3^ cells/well and allowed to adhere for 24 h. Medium containing different concentrations of ACNs-Lipo@CGN was then added, and the cells were incubated for an additional 24 h, with final anthocyanin concentrations of 300 and 500 μg/mL. After treatment, the original medium was discarded, and 100 μL of CCK-8 working solution, prepared by mixing CCK-8 reagent with serum-free medium at a 1:9 ratio, was added to each well. The plates were incubated at 37 °C in the dark for 2 h, after which absorbance was measured at 450 nm. Untreated cells were used as the control group, and blank wells containing only medium and CCK-8 reagent were used for background subtraction. Cell viability was calculated from the absorbance values to evaluate the compatibility of ACNs-Lipo@CGN and determine the safe intervention concentration range.

#### 4.9.2. Establishment of the OA-Induced Lipid Accumulation Model and Intervention

HepG2 cells in logarithmic growth phase were seeded into 6-well plates at 1 × 10^5^ cells/well and allowed to adhere for 24 h. To induce lipid accumulation, oleic acid (OA) was complexed with fatty-acid-free bovine serum albumin (BSA) at a molar ratio of 6:1 (OA:BSA) to prepare a 10 mM OA–BSA stock solution. HepG2 cells were then treated for 24 h with DMEM complete medium containing 0.5 mM OA–BSA complex and 1% fetal bovine serum (FBS). The control groups included a vehicle control, containing BSA at the same concentration as that used in the OA–BSA complex, and blank nanocarrier controls, containing equivalent concentrations of the corresponding anthocyanin-free carriers, Lipo@GA or Lipo@CGN. After model establishment, cells were treated with ACNs control or ACNs-Lipo@GA/ACNs-Lipo@CGN containing different doses of ACNs (low, medium, and high doses; final concentrations determined according to the CCK-8 results) for an additional 24 h. This protocol was designed to evaluate the ability of the nanocarriers to reverse established lipid accumulation.

After intervention, the supernatant was discarded and the cells were washed twice with phosphate-buffered saline (PBS), fixed with 4% paraformaldehyde for 20 min, and stained with Oil Red O working solution prepared in isopropanol for 30 min. Excess dye was removed with 60% isopropanol, followed by PBS washing, and images were captured under an inverted optical microscope to observe intracellular lipid droplets. Intracellular TG levels were quantified strictly according to the TG assay kit instructions and expressed as mmol/gprot.

### 4.10. Statistical Analysis

All experiments were independently repeated three times (*n* = 3), and the data are expressed as mean ± standard deviation (Mean ± SD). For stability data collected at multiple time points, such as thermal stability, photostability, and stability against metal ions and ascorbic acid, one-way ANOVA followed by Tukey’s post hoc test was performed independently at each time point to compare differences among groups. For the in vitro digestion experiments involving sequential stages (SSF, SGF, and SIF), one-way ANOVA was performed independently at each digestion stage, followed by Tukey’s test. All statistical analyses were performed using SPSS 26.0. Differences were considered statistically significant at *p* < 0.05. Figures were prepared using GraphPad Prism 9.0.

## 5. Conclusions

In this study, soybean lecithin was used as the core to systematically compare the differential regulatory mechanisms of two plant polysaccharides with distinct molecular conformations, namely highly branched GA and linear sulfated CGN, in the construction of bilayer nanodelivery systems for ACNs. The results showed that polysaccharide conformation determined the differentiation of interfacial self-assembly behavior and defense mechanisms. The linear, polyanion-rich CGN formed a denser three-dimensional electrostatically crosslinked network with liposomes, thereby endowing the system with a higher encapsulation efficiency (81.87%) and markedly stronger protection against light, metal ions, ascorbic acid, and the harsh simulated intestinal environment, with 51% retention at the targeted intestinal stage. By contrast, highly branched GA showed superior performance under high-temperature thermal processing because of its better interfacial flexibility.

At the level of terminal biological effects, the bilayer nanostructure transformed ACNs from a degradation-prone crystalline state into an amorphous state and significantly enhanced their lipid-lowering efficacy in HepG2 cells, possibly by improving extracellular protection and cellular delivery of ACNs. Among the two systems, the structurally more robust CGN carrier further reduced intracellular TG levels by more than 39% relative to the free ACNs group in a dose-dependent manner. Overall, shell-polysaccharide conformation represents a key design handle for tailoring both the physicochemical stability and physiological efficacy of nanodelivery systems. This study provides a strategy with both theoretical depth and practical value for developing plant-based functional nutritional formulations capable of withstanding complex food-processing conditions and targeting lipid metabolic disorders. Future work should further validate the pharmacokinetic advantages and intervention mechanisms of this bilayer system in in vivo animal models.

## Data Availability

The data that support the findings of this study are available from the corresponding author upon reasonable request.

## Supplementary Materials

The following supporting information can be downloaded at: https://www.mdpi.com/article/10.3390/molecules31101634/s1, Figure S1: 3D response surface plots for multicomponent interactions.
