# Supplementary material for: Conformation-Driven Bilayer Nanocarriers for Anthocyanins Using Shell Polysaccharides: Stabilization Mechanisms and Enhanced In Vitro Lipid-Lowering Activity
Source: Molecules. 2026 May 13;31(10):1634. doi: 10.3390/molecules31101634 (PMC13209688; doi:10.3390/molecules31101634)
Supplement: Supplementary file 1 [file molecules-31-01634-s001.zip › molecules-4294637-supplementary.pdf]

Supplementary Materials

Conformation-Driven Bilayer Nanocarriers for Anthocyanins Using Shell Polysaccharides: Stabilization Mechanisms and Enhanced In Vitro Lipid-Lowering Activity

Chunting Zhu [1,2], Jing Xu [1,2], Yunmei Ma [1,2], Yue Mi [1,2], Xing Yang [1,2], Dongfang Shi [2,*] and Kai Song [1,2,*]

1 School of Life Science, Changchun Normal University, Changchun 130032, China; zct15948840694@163.com (C.Z.); xujingl@yeah.net (J.X.); yunmei0205@163.com (Y.M.); 18097730680@163.com (Y.M.); yangyx0816@163.com (X.Y.)
2 Institute of Innovation Science and Technology, Changchun Normal University, Changchun 130032, China
* Correspondence: shidongfang@ccsfu.edu.cn (D.S.); songkai@ccsfu.edu.cn (K.S.)

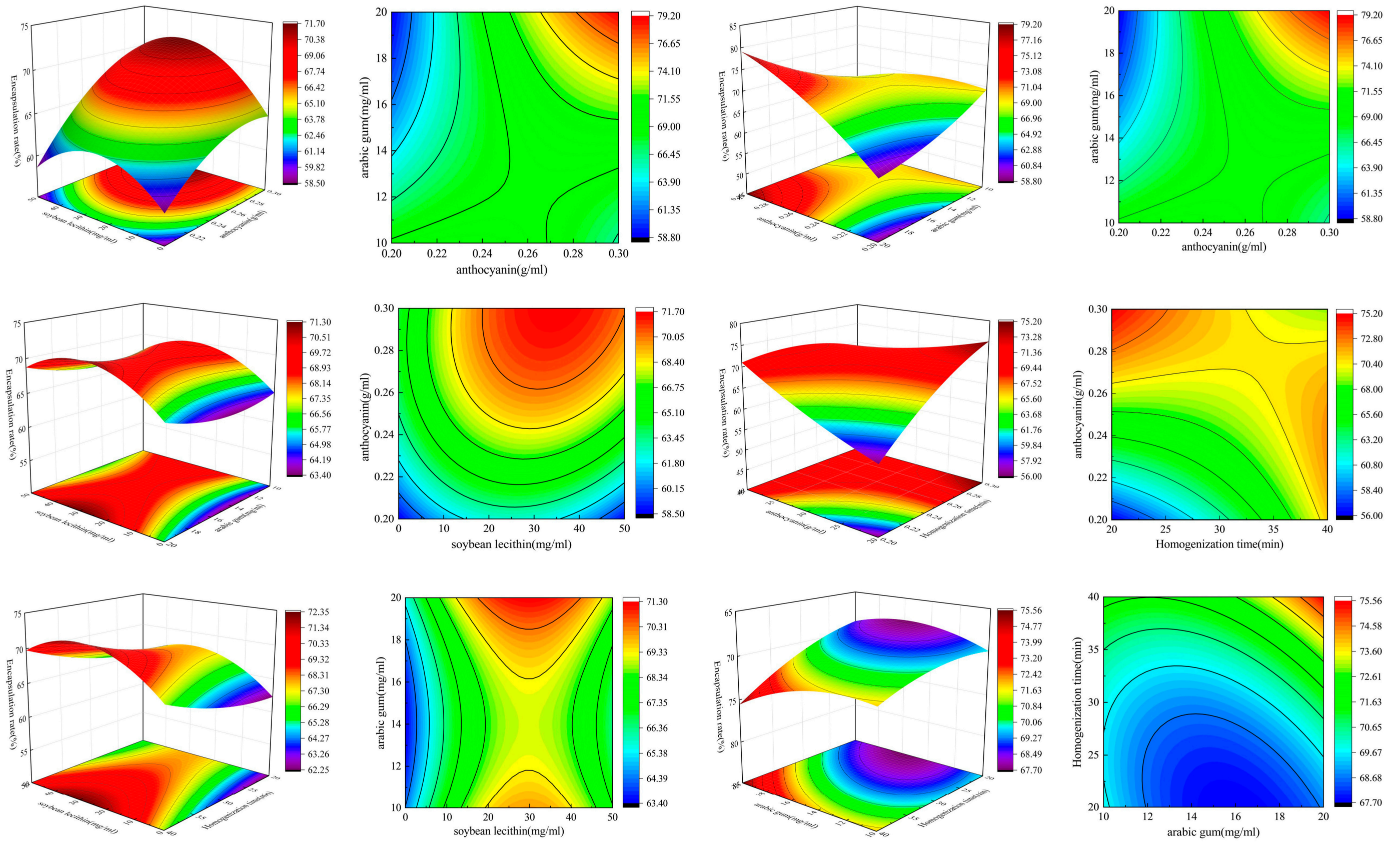

Figure S1. 3D response surface plots for multicomponent interactions.
